# Short-term effects of clown visits in child and adolescent psychiatric care: a pilot study on patient stress and mood outcomes and staff evaluations

**DOI:** 10.3389/fpsyt.2025.1556932

**Published:** 2025-05-13

**Authors:** Amos-Silvio Erik Friedrich, Lorena Holzmeier, Johanna Ehlers, Simone Seebacher, Maggie Rössler, Nadine Skoluda, Urs Markus Nater, Martina Zemp

**Affiliations:** ^1^ Department of Clinical and Health Psychology, University of Vienna, Vienna, Austria; ^2^ Department of Research and Learning, RED NOSES Austria, Vienna, Austria; ^3^ Department of Research and Learning, RED NOSES International, Vienna, Austria; ^4^ Research Platform “The Stress of Life – Processes and Mechanisms Underlying Everyday Life Stress”, University of Vienna, Vienna, Austria

**Keywords:** healthcare clowning, psychiatry, stress, cortisol, humor, nursing, art-based intervention, well-being

## Abstract

**Background:**

Clown visits are an effective intervention to alleviate patients’ stress and anxiety in pediatric hospital settings. However, while children and adolescents in inpatient psychiatric treatment might uniquely benefit from healthcare clowning, little research has addressed the effectiveness of clown visits in this setting.

**Method:**

This pilot study examined the short-term effects of clown visits on psychological (self-reported stress and mood) and physiological (salivary cortisol) outcomes in 29 children and adolescents aged between 7 and 17 years (M = 12.69, SD = 2.90) in a noncontrolled repeated pre-post design over 4 weeks. In addition, 21 care staff members reported separately on their perceived impact of clown visits.

**Results:**

Self-reported stress levels of children and adolescents were decreased significantly from before to after clown visits, whereas salivary cortisol indicated a similar, but nonsignificant decrease. According to the Multidimensional Mood Questionnaire (MDMQ), patients showed significant improvements in energetic arousal, but there were no effects on mood valence and calmness. The effectiveness of the clown visits did not change over repeated visits. Care staff reported that clown visits had a positive impact on patients’ and their own well-being, but their evaluations regarding their stress levels and work processes on the ward were mixed.

**Discussion:**

The present results provide preliminary evidence that the stress-reducing and energizing effects of clown visits, which previous studies have demonstrated in various pediatric settings, can also be transferred to the field of inpatient child and adolescent psychiatry. Mixed self- and work-related evaluations from care staff suggest that improvements in the implementation of clown visits could help eliminate disruptive elements of this intervention.

## 1 Introduction

Hospitalized children and adolescents face a range of challenges, such as loss of control and separation from their home and families, which often lead to high levels of distress (1, 2). Healthcare clowning aims to address the needs of young inpatients for company by creating social encounters that enable humorous coping with negative experiences in the inpatient setting (3, 4). Clowns have particular relevance in pediatric care (5), as it is assumed that children and adolescents respond particularly well to clowning due to the immediate, non-intellectual nature of this approach (4). Previous studies have supported the effectiveness of clown visits for pediatric patients in terms of reducing anxiety, stress, salivary cortisol, pain, and the need for sedation during medical procedures (6–9) as well as improving emotional well-being, energy levels, and mood (10, 11). Humorous reappraisal, as is often applied in clowning, has been shown to be particularly effective in reducing negative affect and arousal associated with adverse events, to an even greater extent than rational reappraisal (12). Indeed, recent meta-analyses have shown particularly strong evidence for improving emotional outcomes such as anxiety (8, 9).

Since dysregulated stress and arousal systems represent a common symptom or maintaining factor across various mental disorders, coping with stress and regulating affect are important transdiagnostic treatment goals in mental healthcare (13, 14). Clown visits are therefore a promising intervention in psychiatric care. However, little attention has been paid to their effects in this setting, except for a small number of pilot studies among adult patients (15–17). A recent study examined the impact of healthcare clowning on adolescents in the inpatient psychiatric setting and found positive effects on self-reported mood and distraction from current problems as well as the perceived atmosphere on the ward (18). The authors noted that future studies would benefit from implementing standardized pre- and post-intervention measures of mood states to further substantiate these findings. Beyond subjective self-report measures, research has begun to investigate the effects of pediatric clown visits on physiological biomarkers, but not yet in psychiatric settings (10).

Notably, healthcare clowning is not only directed at patients but also at medical staff with the aim of improving their working conditions, which are often perceived as stressful and (emotionally) demanding (19). Indeed, previous research has shown that humor can enhance nurses’ mental well-being, improve their energetic arousal and mood, and help them cope with sadness and despair (20, 21). Clown interventions have further been found to improve nursing staff’s interactions (20, 22) and communication (20) with patients. A pilot study in the psychiatric setting reported that care staff generally described healthcare clowning as helpful in their daily routine and supported the continuation of the program, but their evaluations regarding the integration into or disruption of clinic routines were mixed (23).

Taken together, previous research suggests promising avenues for the use of healthcare clowning in inpatient child and adolescent psychiatry, but there is a lack of studies testing its effectiveness in this setting. Thus, the present pilot study aimed to examine how clown visits affect young patients’ self-reported stress and mood levels as well as physiological measures of stress (i.e., salivary cortisol) over four weekly clown visits in psychiatric clinics. Based on past research in other pediatric settings, we hypothesized that following a clown visit, children and adolescents would report improved mood and less stress, and would show reduced cortisol levels compared to before the visit. We further tested whether these effects would be more pronounced over the course of repeated clown visits. Lastly, we were interested in how care staff members perceived the clown visits. Thus, we evaluated the self-reported impact of the clown visits on their own individual mood, the atmosphere on the ward, interactions involving patients, and the patients’ well-being.

## 2 Methods

### 2.1 Study design and procedure

This pilot study used a non-controlled repeated pre-/post-test design. There was no control group and no variation in the treatment; thus, the trial contained only one arm. The study was preregistered at ClinicalTrials.gov (Identifier: NCT04844398). A study protocol outlining the rationale, hypotheses, methods, and procedures according to the SPIRIT 2013 Statement (24) was submitted prior to data collection (25). The study was reviewed and approved by the institutional review board of the University of Vienna (reference number: 00675; date of approval: May 3^rd^, 2021) and by a local ethics committee at one of the participating psychiatric clinics (reference number: 1272/2021; date of approval: November 24^th^, 2021). Data collection took place from November 2021 to August 2022. The self-developed questionnaires used in this study, the analytic code necessary to reproduce the present analyses, and their outputs are openly available at: https://osf.io/zckn6/.

Before and after each visit, we collected saliva samples for the assessment of salivary cortisol, and the children and adolescents completed measures of self-reported stress and mood states before (pre) and after (post) each clown visit on a weekly basis over four consecutive weeks (i.e., four visits in total). Post-assessments were scheduled to take place 20 minutes after the end of a visit in order to capture the delayed cortisol response. Care staff evaluations of the clown visits were assessed within the same time period based on a questionnaire presented to involved staff after each clown visit.

### 2.2 Participants

Participants were recruited at two child and adolescent psychiatric clinics in Austria, which received visits from RED NOSES Austria. The clown visits took place at two different wards of each participating clinic. Children and adolescents of any gender and with any psychiatric diagnoses who were in inpatient treatment during the study period were included in the study. Specific inclusion criteria were (1) age between 7 and 18 years and (2) participation in clown visits at the relevant healthcare facility. Exclusion criteria were (1) potential negative impacts on an individual due to clown visits or study participation, as determined by medical or paramedical care staff, and (2) insufficient command of the German language. Informed consent was obtained from interested participants and their legal guardians before data collection. Recruitment was assisted by care staff on the respective wards. Care staff members were eligible to participate in this study if they were involved with any of the clown visits. This primarily included nurses and therapeutic staff.

This study was preregistered to recruit 50 participants (inpatients) in total. However, recruitment took place during the COVID-19 pandemic and quarantine measures hindered researcher access for extended periods of time. Recruitment was thus concluded after three assessment periods with a total of 31 participants. Of the 31 patients who provided informed consent, two did not participate in any clown visits within the assessment periods and were thus dropped from the analysis leading to a final sample of *N* = 29. In total, 23 responses were obtained from staff members, with two participants contributing more than once. To ensure independence of data, only the first response was considered in the case of multiple assessments, leading to a final sample of *N* = 21 care staff members.

### 2.3 Intervention

A total of 63 clown visits were followed over the course of this study. Clown visits were carried out by teams of two professional clown artists from RED NOSES Austria and took place once a week for four consecutive weeks, in either an individual (75%) or group setting (25%; two to ten patients present). The structure, duration, and content followed standard artistic routines adapted to the current mood and situation of the patients. Routines involved a mixture of rehearsed repertoire and improvisation aimed at actively engaging children and adolescents in humorous play through exaggeration and surprise, absurdity and irrationality, incongruence, and humorous encouragement (4). The mean duration of the clown visits was 16.83 minutes (range: 9 to 35 minutes; median: 16 minutes) in individual settings and 19.40 minutes (range: 10 to 79 minutes; median: 20 minutes) in group settings. Researchers were not present during the clown visits. The clown artists were not involved in any study-related research activities.

### 2.4 Measures

In this report, we focus on the predefined primary outcome measures. Details on other sociodemographic variables, secondary outcomes, and control variables assessed in the study are described in the study protocol (25).

#### 2.4.1 Subjective stress (pre/post)

Patients rated their current stress levels on a visual analog scale (VAS, “Right now I feel stressed”) ranging from 0 (*not at all*) to 100 (*very much*). The VAS approach has been frequently used in previous stress research and has been demonstrated to be sensitive to change (26, 27).

#### 2.4.2 Mood (pre/post)

Patients rated their current mood states across three dimensions (valence, calmness, energetic arousal) using the German short version of the Multidimensional Mood Questionnaire (MDMQ; 28). The MDMQ consists of four mood adjectives per dimension (e.g., “content”, “tired”, “agitated”, “well”) that are rated on a 5-point Likert scale from 1 (*not at all)* to 5 (*very much)*. The measure has shown high validity and reliability in previous research (29). For children aged 11 and younger, we adapted the scale to two items per dimension and added age-appropriate graphic anchors. In the present study, reliability scores (Cronbach’s alpha) were acceptable for the adolescent version (valence: α = .90, calmness: α = .82, energetic arousal: α = .74) as well as for the abbreviated child version (valence: α = .86, calmness: α = .77, energetic arousal: α = .89).

#### 2.4.3 Salivary cortisol (pre/post)

Patients’ saliva samples were collected using SaliCap (IBL Tecan, Hamburg, Germany) kits and analyzed for salivary cortisol as a physiological marker of the autonomic stress response. Under supervision, participants were instructed to accumulate saliva in their mouth for two minutes before transferring it into the tubes using a straw. Tubes were stored at -20°C prior to analysis. Cortisol levels (nmol/l) were measured using luminescence immunoassay (IBL Tecan). Intra- and inter-assay variance was below 10%.

#### 2.4.4 Care staff evaluations (post)

Based on previously applied study tools (5, 30), we developed a questionnaire in order to assess the evaluations of care staff regarding the impact of clown visits on themselves (e.g., individual stress levels, mood, energy levels), the atmosphere on the ward (e.g., general atmosphere, team communication), professional interactions involving patients (e.g., individual attention, affection), and their patients’ well-being (general well-being, course of treatment). Staff evaluated these domains using single items (20 items in total, no psychometric scales) on a rating scale ranging from -2 (*very negative*) to 2 (*very positive*), with 0 as a neutral anchor (*no change perceived*). Staff enjoyment was rated dichotomously (*yes, no, don’t know*). The self-developed questionnaire is freely available at: https://osf.io/zckn6/.

### 2.5 Data analysis

Patient outcomes were analyzed using linear mixed effects models with the *Rstudio* package *nlme* (Version 3.1-165). The majority of patients did not participate at all four time points, mostly due to discharge or changes in treatment schedules. For these cases, data were systematically missing for later time points. Little’s MCAR test confirmed that data were not missing at random (MNAR). The maximum likelihood estimation methods used in this approach are generally well-suited to handle cases with missing data. Deviating slightly from the procedure outlined in the study protocol, we used restricted maximum likelihood estimation (REML) rather than full information maximum likelihood estimation (FIML), as REML has been suggested to be preferrable in the case of small samples (31).

Given the double repeated design (i.e., pre-/post-assessments across multiple clown visits), assessments (level 1) were nested within participants (level 2). While time point was conceptualized as a separate level in the study protocol, we instead opted to include it as a predictor in order to model (dose-response) interactions while maintaining statistical power. Models were built with the predictor variables (1) assessment (coded as 0 = pre, 1 = post) (2), time point (coded as 0–3 for clown visits 1-4), and (3) their interaction (assessment × time point), and selected based on model fit out of models with and without an autoregressive covariance structure as well as with and without random slopes for assessment and/or time point. Modeling random slopes considerably worsened the model fit. We thus retained the more parsimonious fixed slopes models, in which outcomes were modeled with fixed effects of predictors at level 1, a random intercept for participants at level 2, and residual errors following a first-order autoregressive covariance structure. Detailed results concerning model comparisons can be found in **Supplementary Table S1**.

To examine how staff perceived the impact of clown visits in different domains, we descriptively evaluated the distribution of responses (%) using bar charts.

## 3 Results

### 3.1 Participant characteristics

Patients were aged between 7 and 17 years (*M* = 12.69, *SD* = 2.90). The majority identified as female (69%), six as male (21%), and two as diverse (7%), with one missing value (3%). The most common primary diagnoses were eating disorders (37%), mood disorders (20%), and childhood emotional disorders (10%). Most of the children and adolescents (*n* = 16, 55%) were first-time inpatients, while six (21%) had prior admissions (no data on the remaining 24%).

Staff were equally distributed with regard to gender (52% female, 48% male, no other gender identities). With regard to occupational fields, 16 (76%) were nurses, one (5%) was a therapeutic staff member, and four (19%) indicated other fields or roles (e.g., social education worker, nursing intern – no medical staff). Experience in the current professional activity ranged from one month to 26 years (*M* = 49.08 months, *SD* = 73.20, median = 24 months).

### 3.2 Patient primary outcomes

Means and intraclass correlations were estimated based on null (intercept only) models. The mean subjective stress level was *M* = 42.18 (95% CI [31.73, 52.62]) and the mean cortisol level was *M* = 2.48 [3.04, 3.61]. Regarding the mood dimensions, participants showed a mean score of *M* = 3.21 [2.75, 3.68] for valence, *M* = 2.95 [2.58, 3.32] for calmness, and *M* = 3.16 [2.75, 3.57] for energetic arousal. Intraclass correlation coefficients indicated a considerable degree of correlation within patients, at .49 for subjective stress, .46 for salivary cortisol, .67 for mood–valence, .47 for mood–calmness, and .33 for mood–energetic arousal.

**Table 1** shows the results of mixed effects models for the primary outcomes as predicted by assessment (pre-post), assessment time point, and their interaction. The main effect of assessment indicated a significant reduction in subjective stress levels (VAS; *b* = 10.507, *p* = .027) after clown visits compared to pre-assessments. The pre-post effect on salivary cortisol indicated some reduction after clown visits, but with only marginal significance (*b* = 0.718, *p* = .061). With regard to mood states, no significant pre-post effects were found for valence (*b* = 0.220, *p* = .137) or calmness (*b* = -0.037, *p* = .839), but there was a significant improvement in energetic arousal after clown visits (*b* = 0.368, *p* = .008). Time point did not significantly contribute to outcome levels either directly or via its interaction with assessment (pre-post). This finding suggests that the effect of clown visits did not differ substantially between time points.

**Table 1:** Mixed effect models for primary patient outcomes.

Outcome	Fixed Effects					Random Effects				Model Parameters		
	Effect	Estimate	SE	*t*	*p*	Effect	Estimate	95% CI (*SD*)		AIC	BIC	Φ
								Lower	Upper			
Subjective Stress (VAS)	Intercept	46.833	6.361	7.362	<.001^***^	Patient (Intercept)	23.340	15.494	35.161	1150.3	1170.1	.400
	Assessment (pre-post)	**-10.507**	**4.661**	**-2.254**	.027^*^	Residual	24.036	19.042	30.339			
	Time point	-1.851	2.936	-0.631	.530							
	Assessment × Time point	3.982	3.076	1.295	.199							
Salivary Cortisol	Intercept	3.430	0.363	9.461	<.001^***^	Patient (Intercept)	1.301	0.884	1.916	443.4	459.7	-^a^
	Assessment (pre-post)	-0.718	0.378	-1.898	.061^+^	Residual	1.382	1.181	1.616			
	Time point	-0.106	0.179	-0.596	.553							
	Assessment × Time point	0.125	0.241	0.521	.604							
Mood–Valence	Intercept	3.010	0.264	11.414	<.001^***^	Patient (Intercept)	1.149	0.823	1.603	338.3	358.0	.439
	Assessment (pre-post)	0.220	0.147	1.502	.137	Residual	0.782	0.608	1.005			
	Time point	0.110	0.096	1.136	.259							
	Assessment × Time point	-0.034	0.096	-0.350	.727							
Mood–Calmness	Intercept	2.977	0.228	13.071	<.001^***^	Patient (Intercept)	0.818	0.552	1.210	358.7	378.2	.327
	Assessment (pre-post)	-0.037	0.182	-0.204	.839	Residual	0.876	0.717	1.070			
	Time point	-0.005	0.108	-0.049	.961							
	Assessment × Time point	-0.004	0.119	-0.030	.976							
Mood–Energetic Arousal	Intercept	2.916	0.248	11.748	<.001^***^	Patient (Intercept)	0.716	0.201	2.544	329.2	348.7	.728
	Assessment (pre-post)	**0.368**	**0.136**	**2.710**	**.008** ^**^	Residual	1.067	0.619	1.838			
	Time point	0.136	0.107	1.266	.209							
	Assessment × Time point	-0.118	0.089	-1.331	.186							

Assessment was coded as 0 = pre and 1 = post. Time point was coded as 0–3 for clown visits. Salivary cortisol was measured as nmol/l. Analyses are based on 124 (VAS), 112 (cortisol), and 123 (mood) observations, respectively. ^a^No autocorrelation specified, as model fit worsened considerably when included. Significant values are shown in bold. ^+^ *p* <.10. ^*^ *p* <.05.; ^**^ *p* <.01; ^***^ *p* <.001.

Given that multiple outcomes were tested, we controlled for the false discovery rate (FDR), as outlined in the study protocol, using the procedure described by Benjamini and Hochberg (32). The calculations can be found in **Supplementary Table S2**. At an exploratory FDR of 20%, the main effects for energetic arousal and subjective stress remained significant while all other values were above the critical threshold. We thus report original values here, as the interpretation remains unchanged.

### 3.3 Sensitivity analyses

Hospital routines and treatment schedules sometimes interfered with the post-assessment time frame, requiring assessments to be obtained either immediately after a clown visit or more than 20 minutes later. These cases were examined in a sensitivity analysis controlling for deviations from the standardized procedure. We noted these deviations from the design schedule as ‘early post’ for assessments under 10 minutes after clown visits (15 cases) and ‘late post’ for assessments over 30 minutes after clown visits (7 cases). A deviation model was calculated for salivary cortisol levels accounting for deviation as an additional predictor and compared to the final original model. Since we did not expect a similarly delayed response for subjective stress and mood as outcomes, we only considered cases in which post-assessments were taken later than planned to be deviations.

Results of the sensitivity analyses are provided in **Supplementary Table S3**. Models accounting for deviations had a similar fit to the original models. The main effects estimated for assessment (pre-post) did not differ substantially between models. For mood valence, late assessment was associated with a strong additional increase, suggesting further mood improvements in cases when other scheduled appointments might have taken place. For all other models, late and/or early assessment did not exert a significant effect, suggesting that overall, our findings were robust to time deviations of post-assessments. However, these comparisons should be interpreted with caution, as cases of deviation only constituted a small subsample.

### 3.4 Staff evaluations

**Figure 1:**
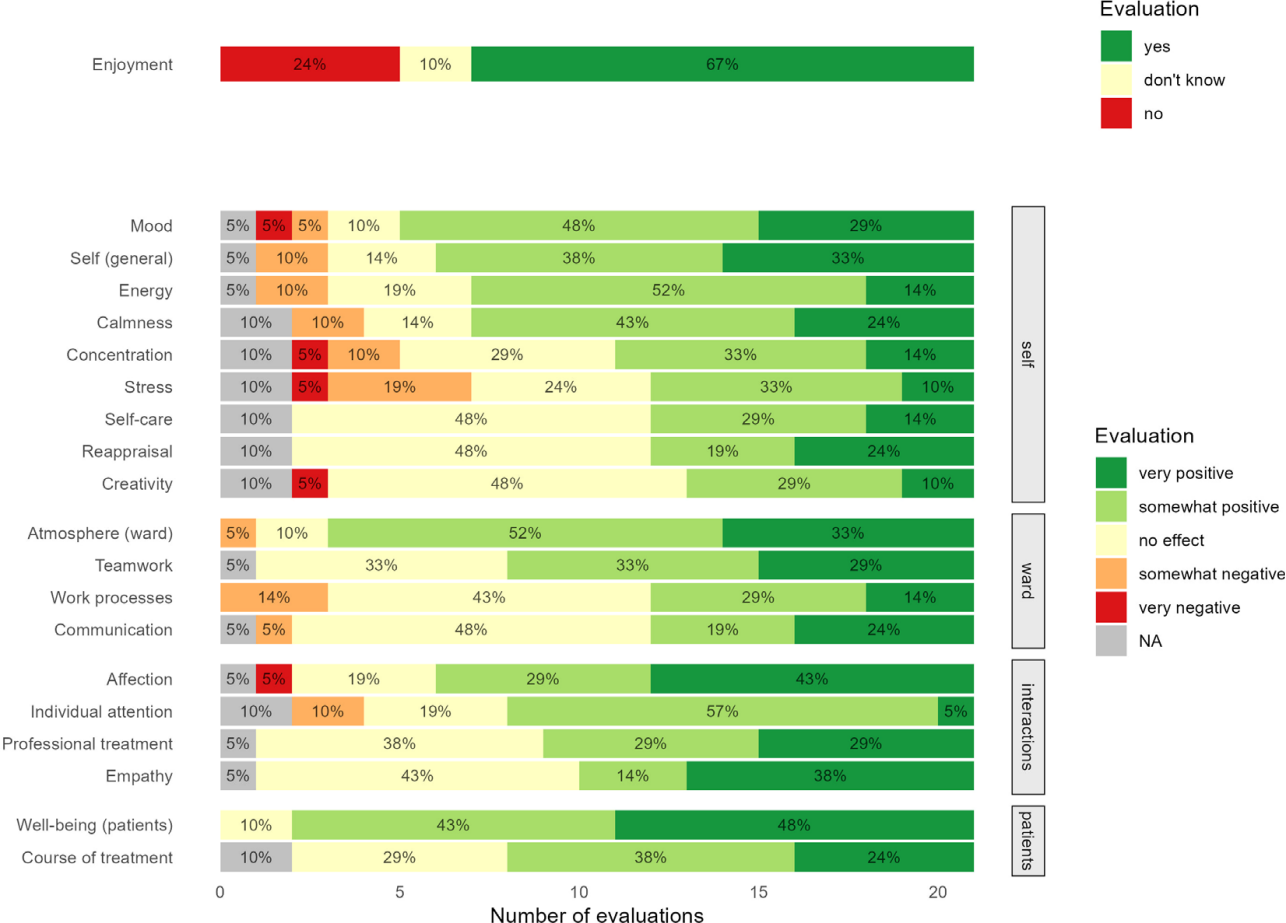
Staff evaluations of the impact of clown visits. NA, Not available.

Staff evaluations of the clown visits were grouped into domains pertaining to the perceived impact on themselves (self), on the atmosphere and processes within the ward (ward), on professional interactions involving patients (interactions), and on patients (patients). An overview of the results concerning staff evaluations (percentages) is presented in **Figure 1**.

Most staff members reported experiencing some positive impact of the clown visits, especially when evaluating the general impact on themselves (*n* = 15, 71% positive) and on their mood (*n* = 16, 77% positive). Evaluations for self-care, change in perspective, and creativity were mainly neutral (*n* = 10, 48% no effect). Negative impacts were sometimes perceived as well, particularly regarding concentration (*n* = 3, 15% negative) and stress levels (*n* = 5, 24% negative).

The majority of care staff members reported a positive impact of clown visits on the general atmosphere on the ward (*n* = 18, 85% positive). Over half of the staff members perceived a positive impact on teamwork (*n* = 13, 62% positive), whereas evaluations of work processes and team communication were more ambivalent, indicating that some staff members perceived disturbances (*n* = 3, 14% negative).

Clown visits were perceived as helpful for professional interactions, specifically regarding affection towards patients (*n* = 15, 72%) and the ability to provide individual attention (*n* = 13, 62%).

Almost all participating staff members reported that the clown visits had positive effects on patients’ well-being (*n* = 19, 90% positive) with no perceived negative effects. The impact on treatment was also rated positively by nearly two thirds of participants (*n* = 13, 62%).

## 4 Discussion

To our knowledge, the present pilot study is among the first to examine the short-term effects of clown visits in child and adolescent psychiatry. The results support the potential of healthcare clowning as a stress-relieving intervention in pediatric psychiatry. Specifically, children and adolescents’ self-reported stress levels were significantly reduced after clown visits compared to before with an average decrease of ten points on the 100-point stress scale, corresponding to a moderate effect size. While our pilot findings are to be interpreted with caution, this is a clinically promising result given that small effects can accumulate with repeated exposure over time (33).

On the physiological level, salivary cortisol levels also indicated a stress reduction, although these effects were not statistically significant in the final model. Beyond low statistical power as a possible explanation for this discrepancy from self-reported stress levels, it is important to note that salivary cortisol is generally only moderately correlated with subjective stress (34, 35), as the measure is affected by a variety of complex neuroendocrine, contextual, and methodological factors (36). Moreover, the finding that cortisol levels did not show a significant change comparable to subjective stress in our study could be due to chronic dysregulation of the hypothalamic-pituitary axis responsible for cortisol regulation, which has been shown to be associated with mental health problems in adults and adolescents (37–39). Given that our study represents the first to include salivary cortisol measures in this context (10), further research may clarify their physiological effect.

On the dimension of energetic arousal, children’s subjective mood showed a significant improvement after the clown visits compared to before, whereas pre- to post-assessments of self-reported mood valence and calmness did not indicate significant changes. Thus, the mood improvements reported in an earlier study are only partially supported in our pre-post design (18). Besides low power in our small sample, these non-significant results might be explained by ceiling effects, as responses were distributed toward the extremes on the valence subscale. Interestingly, individual changes in calmness were rather steep. The overall change close to zero might merely represent a regression to the mean, or it may suggest differential effects depending on individuals’ initial mood levels. Indeed, the clown artists in the present study discussed each patient’s current affective state with care staff during a clinical handover before each visit. During these discussions, different goals were set for the children and adolescents based on their current states, including the intensity level of the intervention.

The repeated pre-post design with up to four clown visits per participant allowed us to investigate possible dose-response relationships, as the (full) effectiveness may only unfold with repeated clown visits. Contrary to our hypotheses, however, the analyses indicated that effects did not change across multiple clown visits. This effect invariance between the time points suggests that clown visits can be just as effective at the first visit or with one single visit as with repeated visits. Accordingly, effectiveness does not appear to depend on the long-term development of the relationship with the artists or on the child becoming accustomed to the intervention. That said, it is possible that our study lacked power to detect changes over repeated visits, as most patients did not participate at all time points. It is also possible that effects change over longer time frames than the four weeks studied, or that they vary by prior familiarity.

The care staff participating in our study provided an additional and important perspective. They confirmed the above results insofar as they evaluated clown visits as exerting a positive impact on patients’ well-being and the course of treatment. Beyond these patient-centered outcomes, staff evaluations were consistent with previous findings in other clinical settings indicating generally positive perceptions. Clown visits were particularly appreciated for their benefits to staff members’ own individual states as well as the general atmosphere on the ward. Given that both of these aspects have shown positive effects on burnout (40), effective communication (41), as well as the experience with and outcomes of inpatient mental healthcare (40, 42, 43), our findings can be seen as clinically relevant. Positive evaluations of work processes and communication among colleagues were less common. Hence, clown visits seem to primarily affect how staff experience their work routines but do little to change how these routines are carried out objectively. Notably, some care staff members even reported negative impacts on work processes, their stress levels, and their ability to concentrate. It is possible that the study procedures contributed to these negative evaluations. However, this is in line with an earlier study in adult psychiatry (23) and might reflect an increased workload due to the (sometimes disruptive) clown visits, as care staff often take on more of a managerial role rather than being a recipient of the intervention. Thus, care staff members might benefit from improvements in the integration of clown visits into clinical routines and could become a more explicit target group within the clown intervention.

### 4.1 Limitations and implications for future research

This study should be regarded as a pilot study yielding preliminary findings in a new field, which comes with some limitations. First and most importantly, we did not include an untreated or active control group. As a consequence, the reported effects cannot be strictly and solely attributed to the intervention. Specifically, we cannot disentangle the specific effectiveness of the clown intervention from general effects (e.g., in relation to increased attention of staff and clown artists, routine treatment elements). To substantiate the present findings, we strongly recommend a controlled efficacy study (i.e., a randomized controlled trial) in order to allow for robust, causal conclusions to be drawn.

Second, high dropout rates due to early discharge of enrolled patients and periods of COVID-19-related quarantines resulted in a high rate of missing data and a smaller sample size than initially preregistered. The small sample size limits the statistical power to detect less pronounced effects. In addition, we only investigated short-term effects, and we cannot derive from our data whether they translate into longer-term effects. Longitudinal data with follow-up assessments over a longer period and with larger samples are needed to elucidate whether our pilot findings can be replicated.

Third, while the present study demonstrated the general feasibility of conducting time-sensitive research on clowning interventions in psychiatric settings, clinical routines and the non-standardized nature of the clown visits often interfered with our rigorously scheduled assessments. While sensitivity analyses revealed that these deviations from the planned study procedure did not significantly influence the main findings, future studies might implement a more flexible design that can be adapted to clinical reality (e.g., continuous inclusion over longer periods, decoupling self-report and saliva collection).

Fourth, model complexity did not allow for the inclusion of person-level variables. We were therefore unable to account for potential moderators that likely affected our results. These may include age and gender, type of mental disorders or symptoms, current medication, individual adaptations of the intervention according to the patient’s current mental state, and familiarity with healthcare clowning, such as the number of clown visits experienced before the start of the study. Indeed, a previous study pointed out the importance of age-appropriateness, i.e., adolescents might respond less positively to clowning than younger children (18). Future studies need to identify which patients benefit particularly from clown visits and whether they are contraindicated for others. This would also enable individualized adjustments to the delivery of clown visits according to specific needs. Moreover, cortisol levels are particularly susceptible to activities that affect physiological states. We assessed potential confounders before each visit (e.g., time since lunch, smoking, sport, caffeine or alcohol consumption) but were unable to control for these due to sample size limitations.

Last, care staff respondents mainly included nurses with few other roles. Future research may clarify whether perceptions generalize across staff members or whether different roles perceive the impact of visits differently.

## 5 Conclusions

While clown visits are an increasingly established intervention in pediatric hospital settings, little research has addressed their implementation in child and adolescent psychiatry. This pilot study provides preliminary evidence for their stress-reducing and energizing effects according to self-reports of children and adolescents in inpatient psychiatric treatment. On a physiological level, salivary cortisol was only slightly and insignificantly reduced following clown visits. The results were not influenced by repeated visits, suggesting that clown visits have an immediate and recurring effect. Care staff reaffirmed patient benefits from their perspective and also perceived positive impacts on their own mood and the general atmosphere on the ward. Overall, our tentative findings reveal promising benefits of clown visits in the field of child and adolescent psychiatry. They underscore the value of interventions that promote play and joy in young patients during potentially stressful inpatient stays.

## Data Availability

The raw data supporting the conclusions of this article will be made available by the authors, without undue reservation.
