# Supplementary material for: Short-term effects of clown visits in child and adolescent psychiatric care: a pilot study on patient stress and mood outcomes and staff evaluations
Source: Front Psychiatry. 2025 May 13;16:1556932. doi: 10.3389/fpsyt.2025.1556932 (PMC12106319; doi:10.3389/fpsyt.2025.1556932)
Supplement: Supplementary file 1 [file Table1.docx]

**Supplementary file S1: Multilevel Model Comparisons for Subjective Stress, Salivary Cortisol, and MDMQ Mood Dimensions**

**Table S1.1**

Model comparisons for subjective stress

|  | **Model 0** | **Model 0a** | **Model 1** | **Model 2** | **Model 3^a^** | **Model 4** |
| --- | --- | --- | --- | --- | --- | --- |
| (Intercept) | 42.282^***^ | 42.176^***^ | 45.055^***^ | 46.833^***^ |  | 47.477^***^ |
|  | (5.249) | (5.263) | (6.210) | (6.361) |  | (5.839) |
| Assessment (pre-post) |  |  | -6.024^+^ | -10.507^*^ |  | -10.553^*^ |
|  |  |  | (3.131) | (4.661) |  | (4.591) |
| Time point |  |  | 0.047 | -1.851 |  | -2.583 |
|  |  |  | (2.545) | (2.936) |  | (3.317) |
| Assessment × Time point |  |  |  | 3.982 |  | 3.880 |
|  |  |  |  | (3.076) |  | (3.017) |
| R2 Marg. | 0.000 | 0.000 | 0.016 | 0.023 |  | 0.013 |
| R2 Cond. |  |  |  |  |  | 0.617 |
| AIC | 1167.8 | 1161.7 | 1154.1 | 1150.3 |  | 1150.8 |
| BIC | 1176.2 | 1173.0 | 1171.0 | 1170.1 |  | 1176.2 |

*Note.* Model 0 = Random intercept model. Model 0a = Random intercept model with autoregressive covariance structure. Model 1 = Model introducing fixed effects of time point and assessment. Model 2 = Model including interaction between predictors. Model 3 = Model including random slope for time point. Model 4 = Model including random slope for assessment.

^a^ Model did not reach convergence.

^+^ *p* < 0.1, ^*^ *p* < 0.05, ^**^ *p* < 0.01, ^***^ *p* < 0.001

**Table S1.2**

Model comparisons for salivary cortisol

|  | **Model 0** | **Model 0a** | **Model 1** | **Model 2** | **Model 3^a^** | **Model 4** |
| --- | --- | --- | --- | --- | --- | --- |
| (Intercept) | 3.044^***^ | 3.043^***^ | 3.365^***^ | 3.430^***^ |  | 3.622^***^ |
|  | (0.285) | (0.285) | (0.340) | (0.363) |  | (0.640) |
| Assessment (pre-post) |  |  | -0.576^*^ | -0.718^+^ |  | -0.264 |
|  |  |  | (0.262) | (0.378) |  | (0.711) |
| Time point |  |  | -0.045 | -0.106 |  | -0.153 |
|  |  |  | (0.134) | (0.179) |  | (0.286) |
| Assessment × Time point |  |  |  | 0.125 |  | -0.115 |
|  |  |  |  | (0.241) |  | (0.381) |
| R2 Marg. | 0.000 | 0.000 | 0.024 | 0.025 |  | 0.011 |
| R2 Cond. | 0.462 | 0.457 | 0.486 | 0.483 |  | 0.499 |
| AIC | 438.6 | 440.6 | 440.7 | 443.4 |  | 529.3 |
| BIC | 446.8 | 451.5 | 454.3 | 459.7 |  | 553.8 |

*Note.* Model 0 = Random intercept model. Model 0a = Random intercept model with autoregressive covariance structure. Model 1 = Model introducing fixed effects of time point and assessment. Model 2 = Model including interaction between predictors. Model 3 = Model including random slope for time point. Model 4 = Model including random slope for assessment.

^a^ Model did not reach convergence.

^+^ *p* < 0.1, ^*^ *p* < 0.05, ^**^ *p* < 0.01, ^***^ *p* < 0.001

**Table S1.3**

Model comparisons for the MDMQ valence subscale

|  | **Model 0** | **Model 0a** | **Model 1** | **Model 2** | **Model 3** | **Model 4** |
| --- | --- | --- | --- | --- | --- | --- |
| (Intercept) | 3.197^***^ | 3.214^***^ | 3.025^***^ | 3.010^***^ | 3.009^***^ | 3.010^***^ |
|  | (0.235) | (0.234) | (0.260) | (0.264) | (0.259) | (0.264) |
| Assessment (pre-post) |  |  | 0.182^+^ | 0.220 | 0.220 | 0.220 |
|  |  |  | (0.098) | (0.147) | (0.145) | (0.147) |
| Time point |  |  | 0.094 | 0.110 | 0.115 | 0.110 |
|  |  |  | (0.085) | (0.096) | (0.103) | (0.096) |
| Assessment × Time point |  |  |  | -0.034 | -0.032 | -0.034 |
|  |  |  |  | (0.096) | (0.096) | (0.096) |
| R2 Marg. | 0.000 | 0.000 | 0.010 | 0.010 | 0.010 | 0.010 |
| R2 Cond. | 0.752 | 0.670 | 0.688 | 0.687 | 0.731 | 0.687 |
| AIC | 337.5 | 327.7 | 333.6 | 338.3 | 341.7 | 342.3 |
| BIC | 345.9 | 338.9 | 350.4 | 358.0 | 367.0 | 367.6 |

*Note.* Model 0 = Random intercept model. Model 0a = Random intercept model with autoregressive covariance structure. Model 1 = Model introducing fixed effects of time point and assessment. Model 2 = Model including interaction between predictors. Model 3 = Model including random slope for time point. Model 4 = Model including random slope for assessment.

^+^ *p* < 0.1, ^*^ *p* < 0.05, ^**^ *p* < 0.01, ^***^ *p* < 0.001

**Table S1.4**

Model comparisons for the MDMQ calmness subscale

|  | **Model 0** | **Model 0a** | **Model 1** | **Model 2** | **Model 3^a^** | **Model 4** |
| --- | --- | --- | --- | --- | --- | --- |
| (Intercept) | 2.948^***^ | 2.950^***^ | 2.979^***^ | 2.977^***^ |  | 2.938^***^ |
|  | (0.183) | (0.184) | (0.221) | (0.228) |  | (0.207) |
| Assessment (pre-post) |  |  | -0.042 | -0.037 |  | -0.023 |
|  |  |  | (0.121) | (0.182) |  | (0.182) |
| Time point |  |  | -0.007 | -0.005 |  | 0.036 |
|  |  |  | (0.092) | (0.108) |  | (0.121) |
| Assessment × Time point |  |  |  | -0.004 |  | -0.010 |
|  |  |  |  | (0.119) |  | (0.118) |
| R2 Marg. | 0.000 | 0.000 | 0.000 | 0.000 |  | 0.001 |
| R2 Cond. | 0.555 | 0.474 | 0.467 | 0.466 |  | 0.618 |
| AIC | 350.0 | 345.1 | 354.3 | 358.7 |  | 358.4 |
| BIC | 358.5 | 356.3 | 371.2 | 378.4 |  | 383.7 |

*Note.* Model 0 = Random intercept model. Model 0a = Random intercept model with autoregressive covariance structure. Model 1 = Model introducing fixed effects of time point and assessment. Model 2 = Model including interaction between predictors. Model 3 = Model including random slope for time point. Model 4 = Model including random slope for assessment.

^a^ Model did not reach convergence.

^+^ p < 0.1, ^*^ p < 0.05, ^**^ p < 0.01, ^***^ p < 0.001

**Table S1.5**

Model comparisons for the MDMQ energetic arousal subscale

|  | **Model 0** | **Model 0a** | **Model 1** | **Model 2** | **Model 3** | **Model 4** |
| --- | --- | --- | --- | --- | --- | --- |
| (Intercept) | 3.213^***^ | 3.163^***^ | 2.950^***^ | 2.916^***^ | 2.910^***^ | 2.936^***^ |
|  | (0.209) | (0.207) | (0.247) | (0.248) | (0.249) | (0.220) |
| Assessment (pre-post) |  |  | 0.236^*^ | 0.368^**^ | 0.364^*^ | 0.369^**^ |
|  |  |  | (0.093) | (0.136) | (0.140) | (0.134) |
| Time point |  |  | 0.084 | 0.136 | 0.147 | 0.143 |
|  |  |  | (0.100) | (0.107) | (0.107) | (0.119) |
| Assessment × Time point |  |  |  | -0.118 | -0.137 | -0.125 |
|  |  |  |  | (0.089) | (0.085) | (0.088) |
| R2 Marg. | 0.000 | 0.000 | 0.013 | 0.015 | 0.014 | 0.015 |
| R2 Cond. | 0.671 | 0.332 | 0.304 | 0.321 | 0.139 | 0.702 |
| AIC | 342.7 | 322.4 | 326.0 | 329.2 | 331.4 | 328.6 |
| BIC | 351.1 | 333.6 | 342.8 | 348.9 | 356.8 | 353.9 |

*Note.* Model 0 = Random intercept model. Model 0a = Random intercept model with autoregressive covariance structure. Model 1 = Model introducing fixed effects of time point and assessment. Model 2 = Model including interaction between predictors. Model 3 = Model including random slope for time point. Model 4 = Model including random slope for assessment.

^+^ *p* < 0.1, ^*^ *p* < 0.05, ^**^ *p* < 0.01, ^***^ *p* < 0.001
