# Supplementary material for: Short-term effects of clown visits in child and adolescent psychiatric care: a pilot study on patient stress and mood outcomes and staff evaluations
Source: Front Psychiatry. 2025 May 13;16:1556932. doi: 10.3389/fpsyt.2025.1556932 (PMC12106319; doi:10.3389/fpsyt.2025.1556932)
Supplement: Supplementary file 2 [file Table2.docx]

**Supplementary file S2: Results of the Benjamini-Hochberg procedure to control for false discovery**

| **Variable** | ***p*** | **Rank (i)** | **B-H value^a^** |
| --- | --- | --- | --- |
| Arousal – Assessment | .008 | 1 | 0.013 |
| VAS – Assessment | .027 | 2 | 0.027 |
| Cortisol – Assessment | .061 | 3 | 0.040 |
| Valence – Assessment | .137 | 4 | 0.053 |
| Arousal – Interaction | .186 | 5 | 0.067 |
| VAS – Interaction | .199 | 6 | 0.080 |
| Arousal – Time point | .209 | 7 | 0.093 |
| Valence – Time point | .259 | 8 | 0.107 |
| VAS – Time point | .53 | 9 | 0.120 |
| Cortisol – Time point | .553 | 10 | 0.133 |
| Cortisol – Interaction | .604 | 11 | 0.147 |
| Valence – Interaction | .727 | 12 | 0.160 |
| Calmness – Assessment | .839 | 13 | 0.173 |
| Calmness – Time point | .961 | 14 | 0.187 |
| Calmness – Interaction | .976 | 15 | 0.200 |

*Note*. ^a^Critical Benjamini-Hochberg values are based on a false discovery rate of .20.
