# Supplementary material for: Short-term effects of clown visits in child and adolescent psychiatric care: a pilot study on patient stress and mood outcomes and staff evaluations
Source: Front Psychiatry. 2025 May 13;16:1556932. doi: 10.3389/fpsyt.2025.1556932 (PMC12106319; doi:10.3389/fpsyt.2025.1556932)
Supplement: Supplementary file 3 [file Table3.docx]

**Supplementary file S3: Sensitivity Analyses**

We noted deviations from the design schedule as ‘early post’ for assessments under 10 minutes after clown visits (15 cases) and ‘late post’ for assessments over 30 minutes after clown visits (7 cases). A deviation model was calculated for salivary cortisol levels accounting for deviation as an additional predictor and compared to the final original model (Table A3.2). Since we did not expect a similarly delayed response for subjective stress and mood as outcomes, we only considered cases in which post-assessments were taken later than planned to be deviations (Tables A3.1 and A3.3-5).

**Table S3.1**

Subjective stress (VAS)

|  | Original model | Deviation model^a^ |
| --- | --- | --- |
| (Intercept) | 46.833^***^ | 46.851^***^ |
|  | (6.361) | (6.362) |
| Assessment (pre-post) | -10.507^*^ | -11.159^*^ |
|  | (4.661) | (5.183) |
| Timepoint | -1.851 | -1.841 |
|  | (2.936) | (2.950) |
| Assessment x Time point | 3.982 | 4.269 |
|  | (3.076) | (3.243) |
| Deviation |  | 2.658 |
|  |  | (9.003) |
| SD (Intercept VPN) | 23.340 | 23.207 |
| SD (Observations) | 24.036 | 24.163 |
| Num.Obs. | 124 | 124 |
| R2 Marg. | 0.023 | 0.024 |
| AIC | 1150.3 | 1146.0 |
| BIC | 1170.1 | 1168.6 |
| RMSE | 20.27 | 20.33 |

*Note.* ^a^The deviation model accounts for deviations (i.e., cases where post-assessments were later than planned) as an additional predictor. ^+^ *p* < 0.1, ^*^ *p* < 0.05, ^**^ *p* < 0.01, ^***^ *p* < 0.001

**Table S3.2**

Salivary cortisol

|  | Original model | Deviation model^a^ |
| --- | --- | --- |
| (Intercept) | 3.430^***^ | 3.425^***^ |
|  | (0.363) | (0.363) |
| PrePost | -0.718^+^ | -0.762^+^ |
|  | (0.378) | (0.456) |
| Timepoint | -0.106 | -0.106 |
|  | (0.179) | (0.181) |
| PrePost × Timepoint | 0.125 | 0.134 |
|  | (0.241) | (0.248) |
| Deviation |  | 0.093 |
|  |  | (0.450) |
| SD (Intercept VPN) | 1.301 | 1.280 |
| SD (Observations) | 1.382 | 1.403 |
| Num.Obs. | 112 | 112 |
| R2 Marg. | 0.025 | 0.025 |
| R2 Cond. | 0.483 | 0.468 |
| AIC | 443.4 | 447.1 |
| BIC | 459.7 | 468.8 |
| ICC | 0.5 | 0.5 |
| RMSE | 1.22 | 1.23 |

Note. ^a^The deviation model accounts for deviations (i.e., cases where post-assessments were earlier or later than planned) as an additional predictor. ^+^ p < 0.1, ^*^ p < 0.05, ^**^ p < 0.01, ^***^ p < 0.001**Table S3.3**

MDMQ valence subscale

|  | Original model | Deviation model^a^ |
| --- | --- | --- |
| (Intercept) | 3.010^***^ | 2.759^***^ |
|  | (0.264) | (0.271) |
| PrePost | 0.220 | 0.214 |
|  | (0.147) | (0.144) |
| Timepoint | 0.110 | 0.176^+^ |
|  | (0.096) | (0.097) |
| PrePost × Timepoint | -0.034 | -0.030 |
|  | (0.096) | (0.095) |
| dev |  | 0.540^**^ |
|  |  | (0.198) |
| SD (Intercept VPN) | 1.149 | 1.114 |
| SD (Observations) | 0.782 | 0.753 |
| Num.Obs. | 123 | 123 |
| R2 Marg. | 0.010 | 0.044 |
| R2 Cond. | 0.687 | 0.700 |
| AIC | 338.3 | 334.4 |
| BIC | 358.0 | 356.9 |
| ICC | 0.7 | 0.7 |
| RMSE | 0.63 | 0.60 |

Note. ^a^The deviation model accounts for deviations (i.e., cases where post-assessments were later than planned) as an additional predictor. ^+^ p < 0.1, ^*^ p < 0.05, ^**^ p < 0.01, ^***^ p < 0.001**Table S3.4**

MDMQ calmness subscale

|  | Original model | Deviation model^a^ |
| --- | --- | --- |
| (Intercept) | 2.977^***^ | 2.973^***^ |
|  | (0.228) | (0.252) |
| PrePost | -0.037 | -0.038 |
|  | (0.182) | (0.182) |
| Timepoint | -0.005 | -0.004 |
|  | (0.108) | (0.113) |
| PrePost × Timepoint | -0.004 | -0.003 |
|  | (0.119) | (0.120) |
| dev |  | 0.009 |
|  |  | (0.226) |
| SD (Intercept VPN) | 0.818 | 0.817 |
| SD (Observations) | 0.876 | 0.882 |
| Num.Obs. | 123 | 123 |
| R2 Marg. | 0.000 | 0.000 |
| R2 Cond. | 0.466 | 0.462 |
| AIC | 358.7 | 361.9 |
| BIC | 378.4 | 384.3 |
| ICC | 0.5 | 0.5 |
| RMSE | 0.75 | 0.75 |

*Note*. ^a^The deviation model accounts for deviations (i.e., cases where post-assessments were later than planned) as an additional predictor. ^+^ *p* < 0.1, ^*^ *p* < 0.05, ^**^ *p* < 0.01, ^***^ *p* < 0.001

**Table S3.5**

MDMQ energetic arousal subscale

|  | Original model | Deviation model^a^ |
| --- | --- | --- |
| (Intercept) | 2.916^***^ | 3.013^***^ |
|  | (0.248) | (0.252) |
| PrePost | 0.368^**^ | 0.391^**^ |
|  | (0.136) | (0.135) |
| Timepoint | 0.136 | 0.088 |
|  | (0.107) | (0.112) |
| PrePost × Timepoint | -0.118 | -0.131 |
|  | (0.089) | (0.088) |
| Deviation |  | -0.482^+^ |
|  |  | (0.285) |
| SD (Intercept VPN) | 0.716 | 0.521 |
| SD (Observations) | 1.067 | 1.152 |
| Num.Obs. | 123 | 123 |
| R2 Marg. | 0.015 | 0.028 |
| R2 Cond. | 0.321 | 0.193 |
| AIC | 329.2 | 329.1 |
| BIC | 348.9 | 351.6 |
| ICC | 0.3 | 0.2 |
| RMSE | 0.90 | 1.03 |

*Note*. ^a^The deviation model accounts for deviations (i.e., cases where post-assessments were later than planned) as an additional predictor. ^+^ *p* < 0.1, ^*^ *p* < 0.05, ^**^ *p* < 0.01, ^***^ *p* < 0.001
